# CAG Student Prize Paper – A6 THE USE OF HLADQA1*05G>A SCREENING FOR THE SELECTION OF NON-TUMOR NECROSIS FACTOR-ALPHA ANTAGONIST ADVANCED THERAPIES IN INFLAMMATORY BOWEL DISEASE: A PROSPECTIVE STUDY

**DOI:** 10.1093/jcag/gwaf042.006

**Published:** 2026-02-13

**Authors:** V Guisandes Bueno, T Ponich, J Gregor, R Khanna, K McIntosh, M Beaton, R Kim, A Wilson

**Affiliations:** Western University Schulich School of Medicine & Dentistry, London, ON, Canada; Western University Department of Medicine, London, ON, Canada; Western University Department of Medicine, London, ON, Canada; Western University Department of Medicine, London, ON, Canada; Western University Department of Medicine, London, ON, Canada; Western University Department of Medicine, London, ON, Canada; Western University Department of Physiology and Pharmacology, London, ON, Canada; Western University Department of Medicine, London, ON, Canada

## Abstract

**Background:**

The HLADQA1*05G>A genetic variation is a clinically-useful screening tool for identifying tumor necrosis factor-α antagonists (TNFA)anti-drug antibody (ADA) risk as well as TNFA loss of response and discontinuation.

**Aims:**

We hypothesize that an additional use for HLADQA1*05G>A screening is guiding the selection of TNFA vs non-TNFA-based advanced inflammatory bowel disease (IBD) treatments.

**Methods:**

A prospective cohort study was conducted in IBD patients being considered for advanced therapy. Participants were assessed in one of 2 cohorts: 1) those where advanced therapy selection was based on physician preference (controls); 2) those where HLADQA1*05G>A screening was used to select the use of TNFA vs non-TNFA advanced therapy (screened cohort). Specifically, TNFA therapy was selected for wildtype genotypes and non-TNFA therapy was selected for variant genotypes. Participants were assessed for clinical remission at 1-year, treatment discontinuation, adverse drug events, ADA formation, and other important clinical outcomes. Post-hoc analyses examined the effect of screening on individual genotype groups.

**Results:**

A total of 233 participants seen at a tertiary care centre in London, ON were included in the final analyses (screened cohort, n = 75; control cohort, n = 158). The distribution of genotypes in the screened cohort was 48 GG, 20 GA, 7 AA. TNFA therapy was the most used advanced therapy in both groups (screened, N = 48/75, 64.0%; controls, N = 84/158, 53.2%). Screened participants were more likely to achieve clinical remission at 1-year vs controls (N = 61/75, 81.3% vs 102/158, 64.6%, p = 0.0093), less likely to be co-prescribed combination therapy (N = 4/75, 5.3% vs 71/158, 44.9%, p < 0.0001), report fewer adverse drug events (ADE) (N = 13/75, 17.3% vs N = 56/158, 35.4%, p = 0.0055) and had a lower frequency of ADA formation (N = 0/75, 0.0% vs N = 11/158, 7.0%, p = 0.018). Similar frequencies of drug discontinuation, hospitalizations, surgery, dose escalation and rescue glucocorticoid use (RGU) were seen in both groups. Post-hoc analyses highlighted that HLADQA1*05G>A variant carriers identified by screening derived the most clinical benefit. Screened variant carriers had the most clinical remission (92.6%) with the fewest ADEs (3.7%), fewest drug dose escalations (11.1%) and requiring less RGU (7.4%) compared to unscreened variant carriers and individuals with a wildtype genotype, with or without screening.

**Conclusions:**

HLADQA1*05G>A-screening was associated with disease remission, fewer adverse events and a lower incidence of ADA formation when used to select TNFA vs non-TNFA therapy. Variant carriers derive the most benefit from screening. We confirm an additional role for HLADQA1*05 screening is guiding TNFA vs non-TNFA IBD drug selection.

**Funding Agencies:**

CIHRAcademic Medical Organization of Southwestern Ontario, Lawson Internal research Fund

